# A computationally inspired in-vivo approach identifies a link between amygdalar transcriptional heterogeneity, socialization and anxiety

**DOI:** 10.1038/s41398-019-0677-1

**Published:** 2019-12-09

**Authors:** Aaron Goldman, Joshua L. Smalley, Meeta Mistry, Harald Krenzlin, Hong Zhang, Andrew Dhawan, Barbara Caldarone, Stephen J. Moss, David A. Silbersweig, Sean E. Lawler, Ilana M. Braun

**Affiliations:** 1 Harvard Medical School, Boston, USA; 2 Division of Engineering in Medicine, Department of Medicine, Brigham and Women’s Hospital, Boston, USA; 3 Department of Neuroscience, Tufts University School of Medicine, Boston, USA; 4 Department of Biostatistics, Harvard T.H. Chan School of Public Health, Boston, USA; 5 Harvey Cushing Neurooncology Laboratories, Department of Neurosurgery, Brigham and Women’s Hospital, Boston, USA; 6 Neurological Institute, Cleveland Clinic, Cleveland, OH USA; 7 Department of Genetics, Harvard Medical School, Boston, USA; 8 Department of Neuroscience, Physiology and Pharmacology, University College, London, UK; 9 Department of Psychiatry, Brigham and Women’s Hospital, Boston, USA; 10 Department of Psychosocial Oncology and Palliative Care, Dana Farber Cancer Institute, Boston, USA

**Keywords:** Molecular neuroscience, Comparative genomics, Depression, Human behaviour

## Abstract

Pharmaceutical breakthroughs for anxiety have been lackluster in the last half-century. Converging behavior and limbic molecular heterogeneity has the potential to revolutionize biomarker-driven interventions. However, current in vivo models too often deploy artificial systems including directed evolution, mutations and fear induction, which poorly mirror clinical manifestations. Here, we explore transcriptional heterogeneity of the amygdala in isogenic mice using an unbiased multi-dimensional computational approach that segregates intra-cohort reactions to moderate situational adversity and intersects it with high content molecular profiling. We show that while the computational approach stratifies known features of clinical anxiety including nitric oxide, opioid and corticotropin signaling, previously unrecognized druggable biomarkers emerge, such as calpain11 and scand1. Through ingenuity pathway analyses, we further describe a role for neurosteroid estradiol signaling, heat shock proteins, ubiquitin ligases and lipid metabolism. In addition, we report a remarkable behavioral pattern that maps to molecular features of anxiety in mice through counterphobic social attitudes, which manifest as increased, yet spatially distant socialization. These findings provide an unbiased approach for interrogating anxiolytics, and hint toward biomarkers underpinning behavioral and social patterns that merit further exploration.

## Graphical abstract

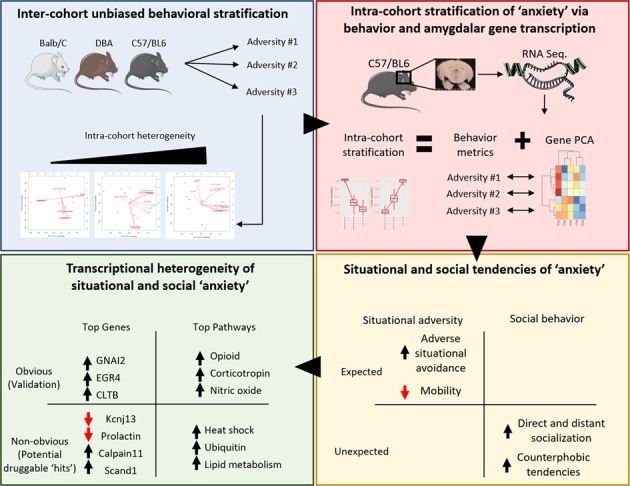

## Introduction

Anxiety disorders have the highest lifetime prevalence of all psychiatric conditions^1^. Despite the ubiquity of these illnesses, few pharmaceutical breakthroughs in the management of acute anxiety have occurred in the last half-century. Existing medications are either slow to work (on the order of weeks to months), incompletely effective (e.g. various classes of antidepressant and buspirone), or plagued by significant risks, from addiction to ataxia to encephalopathy (e.g. benzodiazepines and barbituates). Neuronal circuits in multiple cerebral subregions including prefrontal cortex, hippocampus and amygdala have been implicated in anxiety^2,3^. Of these, the amygdala has emerged as a critical subregion-of-interest in the molecular pathology of anxiety because of its control over fear generation, anxiety-related outputs such as the relationship between hyperexcitability and a link to anxiety in humans, as well as a direct role in suppression of anxiety-like behaviors^4^. A more complete understanding of acute anxiety at the molecular level could inform and lead to the discovery of novel druggable targets^5^. Identifying the molecular features of anxiety could therefore lead to a revolution in rational, biomarker-driven interventions, which should be enabled using models that closely recapitulate the human condition.

The current paradigm in anxiety research relies on in vivo anxiety models that tend to incorporate two components: (1) an animal with an anxious predisposition, usually pathologically, and (2) a measure of situational adversity. With regard to the former component, transgenic mice that manifest anxiety-like behavior via depletion of key neurotransmitters such as serotonin, or genetic traits such as mutations in SHANK3, are popular but tend to demonstrate exaggerated ‘anxious’ behavior as compared to human forms, or do so in the context of unrelated neuropsychiatric disorders including austic behavior^6,7^. As reviewed in depth by Steimer et al., models that invoke ‘fear induction’^8,9^ and/or directed evolution by selective breeding to produce animals with high basal anxiety also imperfectly recapitulate human anxiety^5^. With regard to the situational adversity measure, several ‘anxiety’ challenges are in use today, but many are traumatic such as stress-induced hyperthermia or the forced swim test^5,10^. While a few studies have attempted to elucidate *trait* anxiety using biochemical and biophysical approaches^11^, which may be defined as a baseline anxious phenotype, a comprehensive interrogation of the molecular heterogeneity of cerebral subregions that elucidate how behavior integrates with transcriptional variability remains unexplored.

Here, we sought to address some of the limitations and gaps in pre-clinical approaches for anxiolytics using a rational, computationally inspired model. The goal was to expose molecular and phenotypic heterogeneity in situational anxiety and link these with social and behavior modeling. We aimed to do so without incorporating grossly pathological animals and without exposing traumatizing stimuli. We exposed genetically and developmentally normal isogenic mice to a battery of behavior tests that elucidated ‘anxiety’ by confronting animals with situational adversity choice^12–15^. We carried out high-throughput sequencing on amygdalar mRNA in order to stratify and subsequently pin-point specific genes associated with the elevated ‘anxiety’ condition. In addition to discriminating a cohort of behavioral metrics that stratify transcriptional patterns for human clinical anxiety and ‘anxiety’ in other contexts, we identified 202 highly interconnected genes. Several genes with well-established links to anxiety were present, as well as a number of genes not previously understood to be implicated in anxiety states. While ‘anxious’ mice displayed anticipated situational aversions, they also manifest unanticipated counterphobic social tendencies. These findings suggest a link between transcriptional heterogeneity and social and situational behavior, have implications for methodology by which to study anxiety and hint toward new druggable anxiolytic targets.

## Results

### Mathematical stratification of three isogenic mouse strains based on heterogeneity of behavioral metrics identifies C57/BL6

We first sought to stratify by generalized behavioral heterogeneity among isogenic mouse models, which we hypothesized would later provide a suitable substrate to link molecular mechanisms associated with anxiety, thus revealing inherently diverse murine strains and mitigating the inherent complications of mutations or directed evolution.

**Fig. 1:**
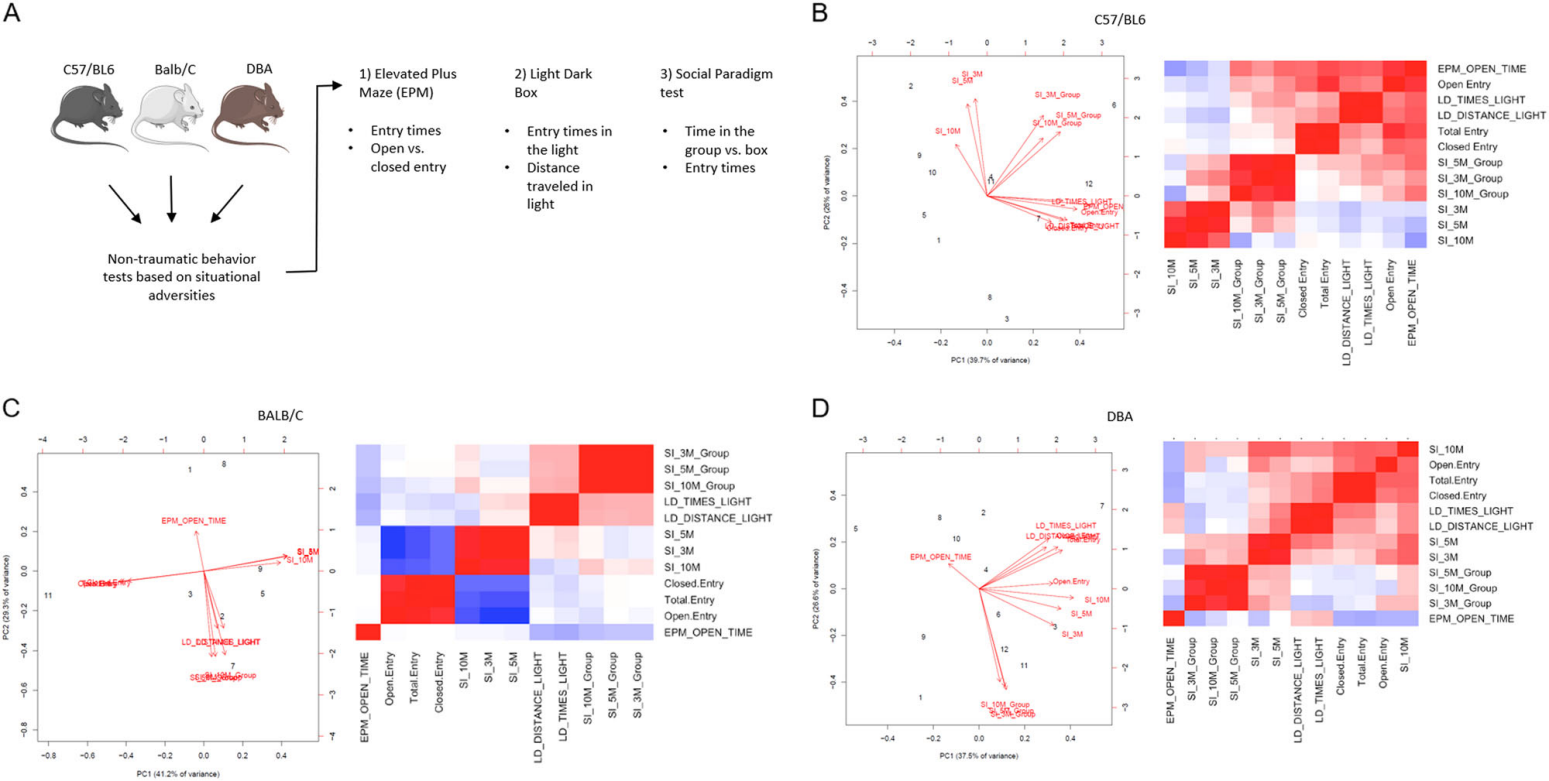
Unbiased mathematical stratification of three isogenic mice strains based on behavioral metrics identifies intra-cohort variability in C57/BL6. **a** Experimental design describes the behavior tests and quantitative metrics deployed to study three isogenic strains of mice, *N* = 12 per cohort. **b**–**d** Centered and standardized principal component analysis (PCA) plots and Pearson correlation matrices segregate the quantitative metrics for elevated plus maze (EPM) entry times into the open or closed arms, social interaction (SI) based on time in the box or group at 3, 5 or 10 min, and the light/dark test (LD) based on time or distance in the light in C57/BL6 (**b**) BALB/C (**c**) or DBA (**d**) mice.

We started with three isogenic mouse strains conventionally used in common research-based animal experiments. Following environmental habituation, age-matched female C57/BL6, DBA/2J and BALB/C mice (*N* = 12 per strain) were exposed to three psychometric tests: the elevated plus maze (EPM), the light/dark box (LD) and the three-chamber social interaction test (SIT), which present the mice with a situational adversity or social challenge, respectively^10,15^ (Fig. 1a). While each of these tests independently represent ‘anxiety-like’ traits^12–15^, and anxiolytics have shown activity in each model including the SIT^16^, our primary intent was to identify strains with inherent diversity of behavior including social cues.

Twelve quantitative parameters from psychometric testing were incorporated into a mathematical strategy, stratifying behavioral metrics using principal component analyses (PCAs). Results from the PCA defined two distinct subgroups of C57/BL6 mice, primarily separated by SIT variables, and secondarily by LD and EPM times, which are visualized in the Pearson correlation matrix (Fig. 1b). In contrast to DBA and BALB/C, C57/BL6 demonstrated a unique degree of variance between phenotypes (as evidenced by the variance carried by PC1 and PC2), as well as unbiased clustering into two clear groups with principal components (Fig. 1c, d). These data suggested that C57/BL6 mice display a clear and defined range of heterogeneity among all cohorts tested.

### Unbiased integration of amygdalar gene transcription and behavior metrics stratify ‘anxiety’ in C57/BL6

We next sought to interrogate the transcriptomic profile of the amygdalae of C57/BL6 mice. Following rapid decapitation (to mitigate induction of stress-induced gene expression profiles), bi-lateral amygdala resections were performed (Supplementary Fig. 1). After confirming the histology of the resected amygdala, isolated messenger ribonucleic acid (mRNA) was subjected to Illumina NextSeq500 sequencing. Initial exploratory analysis established a clear outlier in the data (Supplementary Fig. 2). This sample was omitted and downstream analysis was performed on samples from the 11 remaining mice. Quantitative measures from the behavioral tests for each mouse included: from the EPM, total entries and percentage of time spent on the open arms; from the LD, percentage of time spent in the light and percentage of distance spent in the light; from the SIT, percentage of time spent in the box (i.e. beside the stranger mouse) in the first 3, 5 and 10 min of the experiment.

**Fig. 2:**
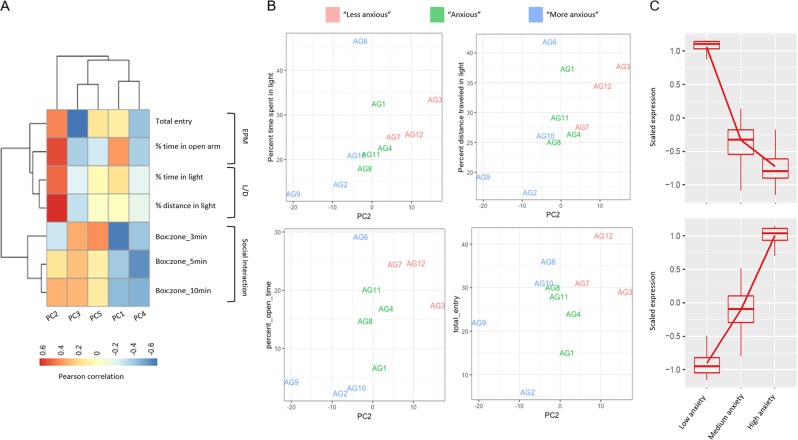
Evaluating the relationship between behavior and the top principal components in a systematic manner. **a** Linear relationship between the first five principal components and psychometric test measures determined using Pairwise Pearson correlation matrix. *N* = 11 mice in C57/BL6 cohort. **b** Principal component analysis shows the relationship between three categories of C57/BL6 based on the separation along the PC2 axis. *N* = 11 mice. **c** Box and whisker plots quantify the scaled expression of genes from RNA-seq from a mice cohort segregated into categories of anxiety based on tertiles of PC2 eigenvalues. *N* = 3 (low anxiety), *N* = 4 (medium anxiety), *N* = 4 (high anxiety).

A PCA was performed, focusing on the top five principal components, which cumulatively explains 90% of the variance in the gene expression data. To evaluate the relationship between behavior and the top principal components in a systematic manner, we computed pairwise Pearson correlations. We determined PC1, which explains 21% of the variance, to be negatively correlated with the SIT measures and PC2, which explains 16% of the variance, to be highly positively correlated with the EPM and LD test measures (Fig. 2a and Supplementary Figs. 3–4). These findings suggested that the first two principal components could be used as a proxy for the behavioral test measures to help simplify the linear model and reduce the risk of overfitting.

Since PC2 correlated well with two of the three behaviors, we used this as our main effect in the linear model and included PC1 as a covariate. Rather than using PC2 as a continuous variable, we separated samples into three groups (“less anxious”, “anxious” and “more anxious”) based on tertiles of the PC2 value (Fig. 2b). We used the likelihood ratio test (LRT) as part of the R Bioconductor package DESeq2 (R Bioconductor package DESeq2, accessed 1/4/19) to identify genes that showed any significant expression change across the three categories of anxiety. At an FDR < 0.05, we observed a total of 209 genes (202 well-characterized) to be differentially expressed (Table S1). Clustering of these genes based on expression levels revealed two prominent expression patterns (Fig. 2c). The majority of significant genes (172) appeared to steadily increase expression with anxiety, and a smaller set (37) exhibited a marked decrease in expression between the “less anxious” and “anxious” group with expression continuing to decrease (although to a lesser degree) moving to “more anxious”.

These results suggest that the phenotypic heterogeneity observed from the behavioral data also manifests at the transcriptional level in the amygdala of an isogenic mouse model. Using simple linear modeling, we were able to incorporate measures from three different anxiety-related behavior tests to identify sets of genes that show significant changes in expression with anxiety.

### Situational adversity avoidance identified in computationally derived ‘anxious’ phenotypes

**Fig. 3:**
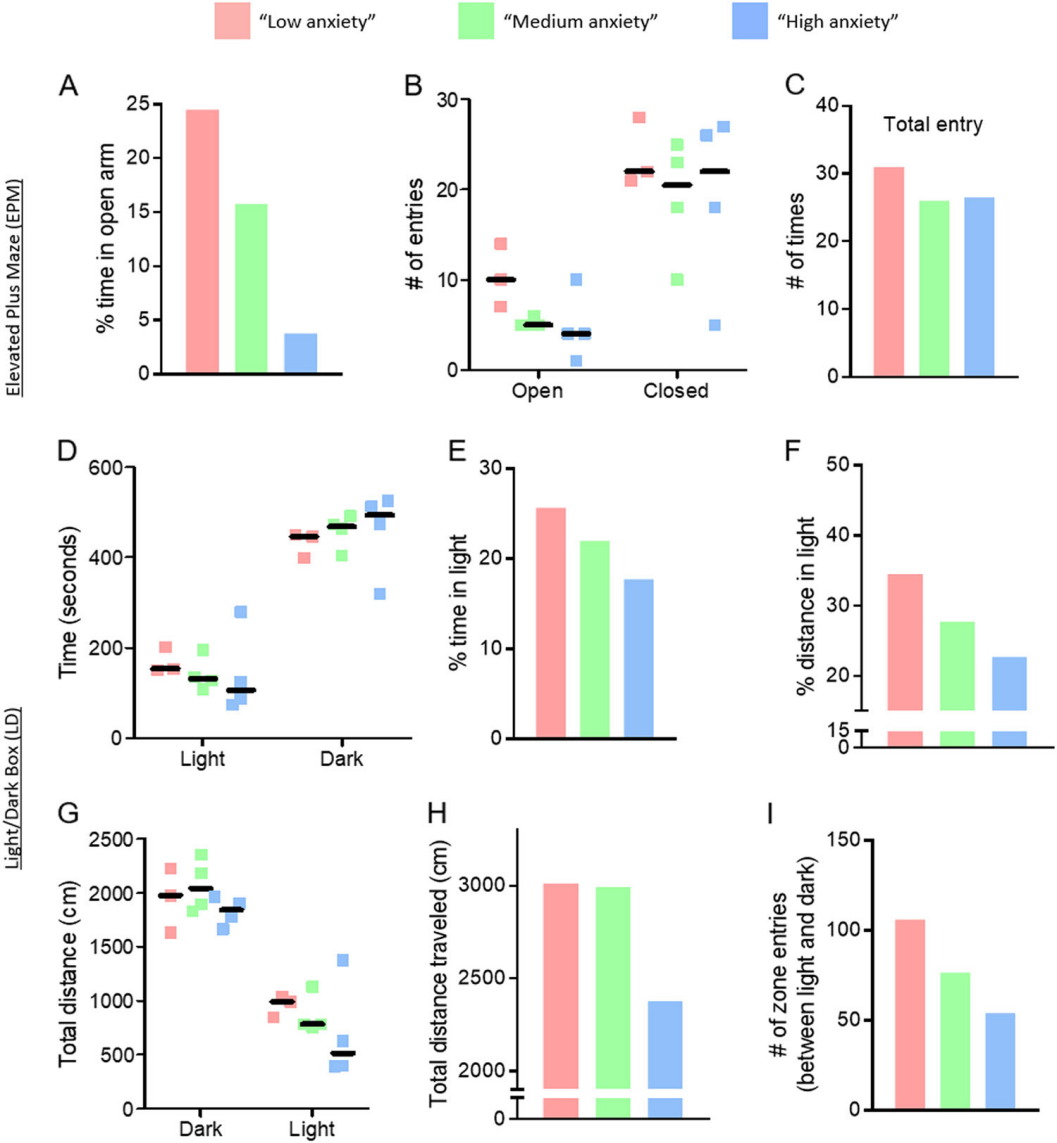
Characterizing situational adversity in ‘anxiety’. Scatter plots show the quantification of EPM metrics (**a**–**c**) and LD metrics (**d**–**i**) for mice categorized in different ranges of anxiety. Hash mark indicates median.

After stratifying mice based on the range of ‘anxiety’ (low, medium and high), we interrogated the data from the LD and EPM tests. ‘Anxiety’-defined features derived from LD and EPM^12^, which were used to classify and segregate mice as described above (i.e. % time in light box, % distance traveled in light box, % time in open arm and total entry from EPM), mapped to the expected phenotype in each cohort (Fig. 3a–f). In validation of our stratification methodology, we determined that ‘anxious’ mice displayed tendencies that were not selected-for by the bioinformatics approach. For example, median distance traveled in both light and dark rooms trended lower in the ‘high anxiety’ vs. medium and low anxiety phenotypes (Fig. 3g, h), indicating an overall stagnation or relative ‘freezing’ of movement, which has tied previously to ‘anxious’ behavior arising from the amygdalae^17^. In accord with these data, median zone entries (i.e., moving between the light and dark rooms) trended lower from low ‘anxious’ mice toward the ‘high anxiety’ phenotype (Fig. 3i).

### Counterphobic social attitude characterizes the behavior of computationally derived ‘anxious’ phenotypes

**Fig. 4:**
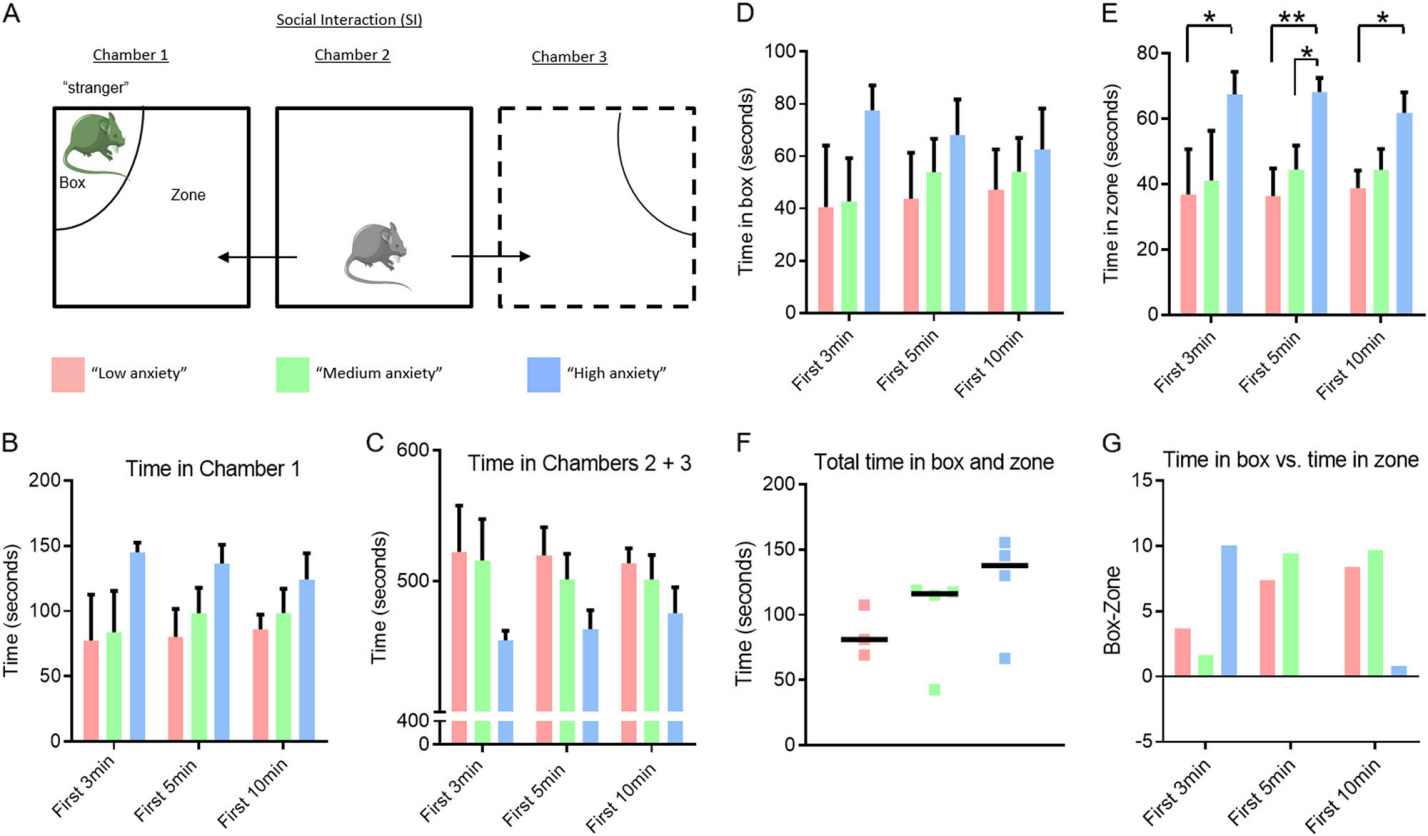
Characterizing social behavior in ‘anxiety’ identifies counterphobic behavior. **a** Schematic illustrates the three-chamber social paradigm test. **b**, **c** Histogram depicts the time spent in the box (**b**) or zone (**c**) in the first 3, 5 or 10 min of the experiment. Error bars indicate SEM. *p < 0.05, **p < 0.01 by *T*-test. **d** Histogram quantifies the total time spent in both the box and zone of each mouse phenotype. **e**) Histogram quantifies the amount of time the mice spent in the box vs. the zone in the first 3, 5 or 10 min of the experiment calculated as the cohort average time spent in the box subtracted from cohort average time spent in the zone.

Next, we interrogated the SIT data after stratifying by ‘anxiety’. The SIT, comprising a three-chamber model in which a previously habituated mouse is introduced to a ‘stranger’ mouse, provides an opportunity for direct (box) or distant (zone) socialization^8^ (Fig. 4a). Indeed, the SIT test is a measure of social anxiety^5,16^, which provides our analysis with greater multidimensionality. Conventionally, anxiety in the SIT paradigm is interpreted as low relative time spent in social contact, i.e., box vs. zone and low overall time with the stranger, i.e., box and zone^18^. In contrast to this convention, we determined that ‘high anxiety’ mice tended to spend relatively more time in the box than other phenotypes over the course of 10 min, preferring also to remain in the ‘stranger’ chamber, as evidenced by significantly longer time in the zone (Fig. 4b, c, *p* < 0.01) and median total-time in both the zone and box compared to other phenotypes (Fig. 4d). However, we determined that while the ‘high anxiety’ mice spent more time in the box vs. zone in the first 3 min than either of the other two phenotypes, this behavior diminished over the course of the 10-min experiment as they remained in the zone longer (i.e., increased distant vs. direct socialization) (Fig. 4e). In contrast, lower ‘anxiety’ phenotypes increased social behavior, as evidenced by a mean increase in the time spent in the box vs. the zone over 10 min (Fig. 4e).

### Validation of intra-cohort ‘anxiety’ via amygdalar transcriptional profiling

**Fig. 5:**
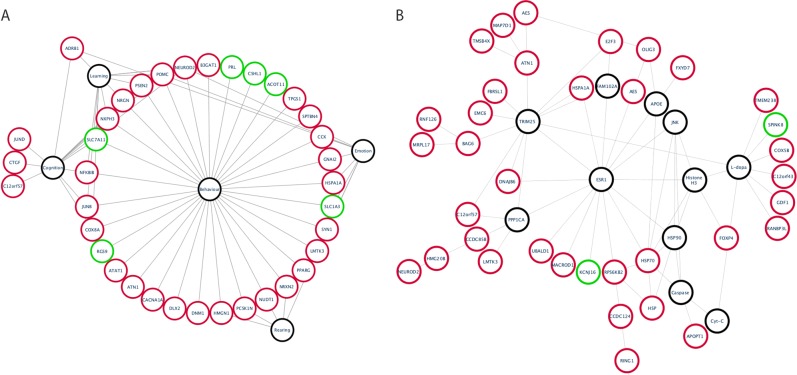
Gene ontology and imputed nodes of ‘anxiety’. The 202 ‘anxiety’ gene signature was analyzed using IPA core analysis. **a** Over-represented gene ontology (GO) terms were identified. The most over-represented term was behavior and its sub-terms: learning, rearing, emotion and cognition. These were assembled into a network diagram showing the terms and the genes associated with each term. Red and green nodes show up- or downregulated genes respectively, black nodes are GO terms. **b** Protein interaction networks were assembled using known interactions in the IPA database. Nodes were imputed that showed two or more interactions with protein products of the genes in the signature. In this way, a large protein network was created. Black nodes are imputed proteins or GO terms whereas red and green nodes show up- or downregulated genes, respectively, from the gene signature.

We sought to test whether the gene expression changes among ‘anxiety’ phenotypes correlated with known behavioral pathways. First, we employed ingenuity pathway analysis (IPA) to perform unbiased interrogations of critical canonical and signaling pathways. Primarily, the most over-represented ontological terms were associated with behavior, emotion and learning (Fig. 5a). Of note, we did not identify ontologies associated with more severe stresses such as restraint stress.

Next, we identified several canonical pathways involving neuron-specific signaling and responses to stress. The most significant pathway identified was the opioid signaling pathway, which frequently associates to anxiety in other contexts^19,20^. This was due to an overall increase in the expression of GNAI2, EGR4, CLTB, RPS6KB2, POMC and CACNA1A (Table S2). To assess the expression of these genes in other subregions, we used freely accessible data available in the Human Protein Atlas (HPA) and quantified the relative gene expression levels of each across multiple regions of the brain in both human and mouse (Supplementary Fig. 5A). Notably, while the HPA mRNA sequencing dataset indicated minimal to no expression of POMC in mouse brain, we queried the Allen Brain in situ hybridization (ISH) dataset, which identified different profiles across the regions of the murine brain (Supplementary Fig. 5B). We also identified pathways involved in nitric oxide signaling, WNT/PCP signaling and corticotropin signaling, which map to known phenotypes in anxiety^21–23^.

### Novel transcriptional profiles as putative druggable targets identified in ‘anxiety’

In addition to the evidence above—that known biological pathways and ontologies associated with state anxiety, which validated our overarching approach to stratify isogenic mice based on a cadre of behavioral metrics integrated with RNA transcription—we also pin-pointed novel changes in gene transcription that may serve for future interrogation as novel biomarkers. Of note, the highest magnitude of upregulated gene expression changes in the ‘anxious’ cohort implicated calpain 11 (CAPN11) and scan domain containing 1 (SCAND1) together with PPARG, which implicate potential druggable targets in calcium-dependent proteases^24^, and adipogenesis and insulin sensitivity arising from zinc finger domain activation^25,26^, respectively. Conversely, the most downregulated genes observed were KCNJ13 and prolactin (PRL) in the mice that clustered in the high ‘anxiety’ phenotype (Table S1), which implicate potassium voltage-gated family and oxytocin stimulation^27^.

Next, we used a combination of IPA and Cytoscape to self-assemble protein networks allowing the programs to impute nodes that link genes in the gene signature (Fig. 5b). In this way, we were able to study the known interactions between the genes in the gene signature and create a large interaction network. Nodes within the dataset included HSP70, ATN1, RPS6KB2 and E2F3, which were significantly upregulated in anxious mice and formed important nodes in the protein network (Fig. 5b). Imputed node proteins were identified in the heat shock, ubiquitin and lipid metabolism families such as TRIM25, PPP1CA, ESR1, HSP90, FAM102A, APOE and JNK.

To provide translational impact, we queried the latest build of the Connectivity Map (cMap) with the ‘anxiety’ signature that we identified, which we previously demonstrated can help link neurological diseases with potential therapeutics^28^. The cMap compares a query gene signature with a database of chemical- and knockout-induced gene expression profiles. In this way, physiological relevance can be inferred by gene expression profile similarity. The gene signature showed significant similarity with gene expression profiles for drugs that regulate opioid signaling (loperamide), the dopaminergic system (verapamil and loxapine) and the adrenergic system (carvedilol, mirtazapine, nadolol, desipramine, labetalol, bisoprolol, sotalol) (Table S3).

Taken together, these evidences resolved a robust and highly interconnected set of genes from ‘anxious’ mice. These genes are highly enriched for known behavioral modulators and significantly identify several important anxiety-related cellular pathways, validating our mathematically derived anxiety platform and identifying potential druggable anxiety targets.

## Discussion

The pharmacologic armamentarium to manage acute anxiety is far from ideal. Antidepressants take weeks to reach maximal effect, thus remain suboptimal in the acute setting. By contrast, barbiturates and benzodiazepines work rapidly, but are rife with risks: encephalopathy progressing to irreversible dementia, ataxia leading to falls and other injuries, and addiction, increasing risk for overdose. One factor in the slow pace of anxiolytic pharmaceutical development is a relative lack of appropriate pre-clinical models, from petri dish to small animal. The efforts described herein merged unbiased, computational methodologies with biological insights to develop a small animal behavior platform for anxiolytic drug development. In the process, this research project has yielded novel insights regarding: (1) ‘anxious’ murine social behavior and (2) molecular heterogeneity of the murine amygdala in ‘anxiety’.

Our research gave shape to a mathematically inspired model of murine anxiety addressing some limitations in extant models (e.g., knock-out mice or mice strains produced through directed evolution) including: ‘anxiety’ behavior that is exaggerated as compared to human behavior, or comorbid with other disorders, for instance ‘autism’. To do this, we measured basal anxiety in three isogenic mouse strains (C57/BL6, DBA/2J and BALB/C) and identified C57/BL6 as exhibiting the greatest range in ‘anxiety’ phenotypes. These results confirm those of other laboratories, as well as an emerging hypothesis in the literature that isogenic mice manifest significant inter- and intra-strain differences in ‘anxiety’^29^. We subjected C57/BL6 mice to ‘anxiety’-provoking stimuli and employed mathematical modeling to identify behavioral assays and metrics that best distinguish ‘anxious’ and ‘non-anxious’ mice^29,30^. By design, our ‘anxiety’ measure battery excluded traumatic stimuli such as stress-induced hyperthermia^31,32^ or challenge with a snake^33^. We believe that our ‘anxiety’ model based on three tests of behavior, and associated psychometric parameters, has wide-ranging applicability in situational anxiety management and may prove useful in in-vivo testing of anxiolytic compounds.

In the process of refining this new murine model for ‘anxiety’, we arrived at a more nuanced understanding of murine ‘social anxiety’ than currently exists in the literature. Classic interpretations of SITs assume greater ‘anxiety’ as time spent engaging in social behavior decreases^34^. To the best of our knowledge, however, SITs have rarely been coupled with other anxiety measures in experiments. When we did couple ‘social anxiety’ measures with other ‘anxiety’ measures, we found that mice who score in the ‘anxious’ range on other ‘anxiety’ measures spend more time, not less, with a stranger mouse. While our experimentation was not designed to elucidate why this might be the case, ethologically relevant interpretations can be made, for instance, counterphobic social tendencies or a desire “to keep [potential] enemies close”. Additional experimentation is necessary to investigate further.

On a molecular level, our analyses identified several novel findings deserving of further exploration. We detected upregulation of several genes associated with synaptic plasticity: NXPH3, NRGN and NRXN2. NXPH3 is expressed in glutamatergic neurons and is thought to tune excitatory synaptic transmission^35^. NRGN is a schizophrenia risk gene and fragile-X mental retardation protein (FMRP) target that is involved in memory formation^36^. NRXN2 is a schizophrenia and autism risk gene that is important for determining synaptic shape^37^. We used network analysis to identify major nodes that connect a number of changed genes that we detected and identified ESR1, the gene that codes for the estrogen receptor α protein. ESR1 is highly expressed in the amygdala and mediates, in part, the cellular responses to neurosteroid estradiol (neuronally synthesized estradiol). Estradiol has been shown to have profound effects on neuronal excitability and synaptic structure^38,39^. Taken together, these data indicate that differences in neurosteroid estrogen levels may play a role in the basal levels of anxiety in mice by mediating physical changes in synaptic structure and therefore neuronal excitability.

Estradiol also modulates the production of other hormones, such as prolactin^40,41^. In our experimentation, downregulation of the prolactin gene was observed in the murine amygdala during stress. Prolactin, a hormone tied to pregnancy and breast-feeding, is generally thought to be peripherally upregulated in the anxious state; however, this finding is tied to pituitary release. Prolactin’s function in the amygdala is uncertain^42^. One hypothesis is that this neuropeptide functions as an anxiolytic in regulation of the neuroendocrine stress axis during the peripartum period^42^. Given that our study exclusively relied on nulliparous female mice, replicating the experiment in pregnant females and male rodents is necessary and may reveal important sex and developmental differences in physiologic reactions to stress.

Finally, a G-protein coupled receptor (GPCR), ADGRL4, is significantly upregulated in the anxious murine state. Interestingly, the ADGRL4 gene has been shown to be estrogen responsive^43^. This receptor is known to be associated with angiogenesis^44^ but until now has no known connection with anxiety states. Our early experimentation suggests that ADGRL4 agonism might hold utility in the management of acute anxiety. Future research endeavors should investigate the activity of this GPCR in subregions of the amygdala as compared to other control regions of the mouse brain.

The results described in this study suggest further investigation of the target genes to understand the functional role of proteins identified from the transcriptional profile, and the amygdalar subregions in which they function, is warranted. Moreover, the relative contribution of anxiety-related genes, identified here, should be understood across other subregions of the brain using similar molecular interrogation approaches. Nonetheless, this work describes a group of effective assays to identify mice with high baseline anxiety that may serve as a representative model of trait anxiety.

## Methods

### Animals

This research used age-matched female C57BL/6, DBA/2J and BALB/cJ mice (*N* = 12 per strain). Animal behavior tests were performed on a single occasion. No specific statistical test was performed to determine sample size. Rather, consultation from bioinformaticians and other experts in the field were considered in order to arrive at a sample size that would allow for reasonable stratification of mice into unique cohorts, and subsequently allow for reasonable statistical measurement of gene transcriptional differences. Mice were introduced to behavior testing (described below) in random order. Sequencing analyses were performed in a single batch. Mice were purchased from Jackson Labs at 7 weeks and allowed to acclimate to the laboratory environment for 2 weeks prior to testing. At 9 weeks of age, they were exposed to the EPM; at 10 weeks, the black/white box; and at 11 weeks, the three-chamber social interaction experiment. Mice were allowed at least 20 min to acclimate to each testing environment, which was carefully engineered to be quiet and free of distraction. In all cases, where necessary and possible, the ‘operator’ was ‘blinded’ to the treatment conditions. The three experimental conditions are explained in greater detail below. All in vivo experiments were performed in compliance with IACUC protocol approved through Harvard Medical School and Brigham and Women’s Hospital, and in accordance with institutional guidelines, supervised on-site by veterinary staff.

### Behavior tests

#### Elevated plus maze

This experimental model was used to assess emotional behavior and levels of general anxiety in mice. The EPM consists of two open and two closed arms extended out from a central platform. A camera, mounted from the ceiling, allowed the experimenters to observe murine behavior from outside the testing chamber. A computer-assisted video-tracking system (TopScan software, CleverSys Inc.) was used to record the number of open and closed arm entries as well as the total time spent in the different maze compartments over the course of 5 min. The percentage of time spent in open arms was used as a surrogate measure for anxiety-like state using the following formula: (open/open + closed) × 100. The EPM was performed under bright light. The start location of the EPM was on an open arm (around mid-way up the arm) with the mouse facing toward the center of the maze.

#### Light/dark box

Developed by Crawley and Goodwin^45^, the LD box highlights the ethological conflict between an animal’s desire to explore a new environment, and yet remain shielded from predators. Our apparatus consisted of two equally sized chambers, one dark and one brightly illuminated (700–800 lux), with a small opening connecting the two (Med Associates Dark Box Insert for Mouse Open Field Activity, Product Number: ENV-511). A mouse was allowed over 10 min to move freely between chambers and the number of entries into the bright chamber and the duration of time spent there was used as indices of bright-space anxiety in mice. Note: the experiment is initiated with the mouse in the light side of the box.

#### Three-chamber social interaction

A three-chambered rectangular apparatus (62 cm × 40 cm) made of clear plexiglass was used to evaluate social preference. Test mice were first placed in the middle chamber and allowed to explore all three chambers for 10 min. After this habituation period, the test mouse was confined to the center chamber while an unfamiliar female mouse of the same strain, that had no prior contact with the subject mouse, was confined to a random, counterbalanced side chamber in a small wire cup. The test mouse was then allowed to explore the entire test apparatus for a 10-min session. The amount of time spent in each chamber and the time spent in close proximity to either the empty cup or the cup with the stranger mouse, was scored by an automated video-tracking system (TopScan software, CleverSys Inc., Reston, VA). Decreased duration spent near the stranger mouse as compared to the empty cup was used as a measure of social anxiety.

The EPM is used to assess general anxiety levels in mice. The LD highlights the ethological conflict between an animal’s twin desires to explore new environments while also remaining shielded from predators. The SIT test evaluates social preference.

### Stratification of isogenic mouse strains based on 12-parameter behavior model

Four parameters were defined from the EPM experiment: (1) the time in seconds spent in open arm of EPM (EPM Time in Open Arm); (2) the number of times a mouse entered the open arm of EPM (EPM Open Entry); (3) the number of times a mouse entered the closed arm of EPM (EPM Closed Entry); and (4) the number of times a mouse entered either arm of EPM (EPM Total Entry). Two parameters were defined from the black/white box include the percentage of total distance traveled spent in the lighted portion of the box (B/W Distance Traveled in Light) and the percentage of total time spent in the lighted portion of the box (B/W Time Traveled in Light). The six parameters from the social interaction (SI) experiment included: SI_3M (first 3 min in the box), SI_5M (first 5 min in the box), SI_10M (first 10 min in the box), SI_3M_Group (first 3 min in the group), SI_5M_Group (first 5 min in the group) and SI_10M_ Group (first 10 min in the group).

For each mouse subtype, we first analyzed the distributions of the values for each of the metrics integrated to identify those that manifest bimodal distributions (suggesting an ability to differentiate between mice with ‘anxiety’ behaviors and mice without). Next, each of the data points was centered and standardized to zero mean and unit variance, using a *z*-score transformation. For each of the mice subgroups, principal components were obtained. These represent the linear combinations of metrics that best describe the variability between the individual mice. We sought to find, across all metrics we considered, the strongest differentiators of phenotype, with equal weighting to all parameters. Moreover, to facilitate this, prior to implementing the principal components analysis, all data from all psychometric testing was standardized to mean 0 and variance 1 to ensure comparability at the same scales; it was after this analysis that the SIT emerged as a key differentiator in psychometric phenotype. That is, when performing analysis for PCA to identify mouse subgroups, we were careful to define principal components in an unbiased manner so that downstream analyses would not be biased by our group stratification. After ensuring that the first two principal components carried the majority of the variance between the individual mice with respect to these metrics, we were able to show a clustering into two distinct groups of mice. This was used to define the mice with behaviors more representative of “anxiety,” as opposed to those with behaviors less representative of “anxiety”. To ensure that the principal components we used for the separation into subgroups were in keeping with our initial hypotheses of how anxiety-like behaviors would manifest, we visualized the original variables in the principal component space. This facilitated an understanding of which results of psychometric testing were more discriminatory between the anxiety-like behaviors of the mice.

### Neurosurgery

An Institutional Animal Care and Use Committee-approved rapid decapitation procedure was employed in the absence of neurological sedatives, carbon dioxide suffocation or anesthesia. A trained neurosurgeon isolated amygdala following a stereotaxic dissection protocol that harnessed murine brain geometry. Amygdala was defined according to a mouse stereotaxic atlas. A dissection microscope was employed for the dissection. Brains were gently removed from the skull and stripped of the meninges. Next, brains were submerged in RNAase-free phosphate-buffered saline and mounted on a vibratome. Cuts were made at bregma −1 mm and −2.75 mm. The resulting slice was then placed with the caudal aspect facing upward. Areas identified as amygdala were removed (1–3 mm lateral from the midline and 0–1 mm from basal to apical). Bilateral amygdalar Vibratome 1000-sliced biopsy tissue was homogenized, bathed in RNAase-free phosphate-buffered saline, and centrifuged. The precipitate was then extracted and snap frozen in liquid nitrogen. The tissue was placed in Trizol (Thermo Fisher). Ribonucleic acid was extracted using the phenol–chloroform phase separation and prepared for subsequent RNA Library preparation.

### RNA Library preparation and sequencing

RNA libraries were prepared using Illumina TruSeq Stranded mRNA sample preparation kits from 500 ng of purified total RNA according to the manufacturer’s protocol. The resultant dsDNA libraries were quantified by Qubit fluorometer, Agilent TapeStation 2200, and RT-qPCR using the Kapa Biosystems library quantification kit according to manufacturer’s protocols. Uniquely indexed libraries were pooled in equimolar ratios and sequenced on a single Illumina NextSeq500 run with single-end 75 bp reads by the Dana-Farber Cancer Institute Molecular Biology Core Facilities.

### RNA-Seq read mapping and expression level estimation

All samples were processed using an RNA-seq pipeline implemented in the bcbio-nextgen project (https://bcbio-nextgen.readthedocs.org/en/latest/). Raw reads were examined for quality issues using FastQC (http://www.bioinformatics.babraham.ac.uk/projects/fastqc/) to ensure library generation and sequencing data were suitable for further analysis. If necessary, adapter sequences, as well as other contaminant sequences such as polyA tails and low-quality sequences were trimmed from reads using cutadapt^46^. Trimmed reads were aligned to the UCSC build mm 10 of the mouse genome using STAR^47^. Quality of alignments was assessed by checking for evenness of coverage, rRNA content, genomic context of alignments, complexity and other quality checks. Expression quantification was performed with Salmon^48^ to identify transcript-level abundance estimates and then collapsed down to the gene-level using the R Bioconductor package tximport^49^.

### Statistical analyses and classification of anxious mice

Statistical analysis was performed using Prism software (GraphPad) determined by two-tailed Student’s *t*-test used to identify statistical significance between individual groups with similar variance.

For initial exploratory analysis, filtering was applied to the full normalized counts matrix. Low expressers (bottom 25% based on average expression) were removed and of those only the most variable genes (the upper quartile based on coefficient of variation) were retained. PCA was used to identify sample AG5 as a clear outlier (Supplementary Fig. 2). This sample was removed and downstream analyses were performed on the remaining samples using all 47,729 genes in the original counts matrix.

To tie together the behavioral data to the molecular phenotypic variability data, correlations were computed for all psychometric test metrics against the first five principal components from the PCA post-outlier removal. The variables incorporated include: from the EPM, total entries and percentage of time spent on the open arms; from the black/white box experiment, percentage of time spent in the light and percentage of distance spent in the light; from the three-chamber social interaction experiment, percentage of time spent in the box beside the stranger mouse in the first 3, 5 and 10 min of the experiment. Based on a high correlation of PC2 with several of the metrics (LD and EPM), we used PC2 as a proxy for these metrics in our model fit. Rather than using PC2 as a continuous variable, we separated values in tertiles to classify groups of “less anxious”, ‘anxious’ and “more anxious”. Additionally, because we observed PC1 to explain 21% of the variance in the data, and observed slight negative correlations with the box:zone ratios, we included it as a covariate in the model to control for this effect.

Differentially expressed genes were identified using the LRT as part of the R Bioconductor package DESeq2 (ref. ^50^). Significant genes were obtained using an FDR threshold of 0.05. Genes were separated into clusters based on similar expression profiles across the defined anxiety groups.

### IPA, cytoscape and cMap analysis

Differentially expressed genes were used to create an ‘anxiety’ gene signature of 202 well-characterized genes as described above. This was analyzed using core IPA to identify the involvement of biological pathways in an unbiased manner. Gene lists were matched to the IPA curated database of canonical signaling pathways and used to detect significantly represented biological pathways (*p* < 0.05). Next, the individual genes were matched to their ontological terms using IPA. The most over-represented ontologies were identified (behavior, emotion, learning, rearing, cognition) and protein network diagrams showing the genes for each of these ontological classes were created using Cytoscape. IPA was then used to build networks of known interactions between the protein products of the differentially expressed genes in the signature. IPA was allowed to impute highly connected protein nodes that connected directly to genes present in the gene signature in order to create a large protein network. The subsequent network was visualized in Cytoscape. Finally, we used the 202 gene ‘anxiety’ signature to query the latest version of the Connectivity Map (cMap) (https://clue.io/). Comparisons were carried out according to refs. ^51,52^ and the top 15 perturbagens with the most matching profiles were selected.

## Supplementary information

Supplemental Figure Legends

Supplemental Figure 1

Supplemental Figure 2

Supplemental Figure 3

Supplemental Figure 4

Supplemental Figure 5

Supplemental Table 1

Supplemental Table 2

Supplemental Table 3

## Data Availability

RNA sequencing files can be accessed in the GEO Accession database under the ID: GSE132457 (https://www.ncbi.nlm.nih.gov/geo/query/acc.cgi?acc=GSE132457).

## References

[1] Bandelow B, Michaelis S (2015). Epidemiology of anxiety disorders in the 21st century. Dialogues Clin. Neurosci..

[2] De Bellis MD (2000). A pilot study of amygdala volumes in pediatric generalized anxiety disorder. Biol. Psychiatry.

[3] Gross C, Hen R (2004). The developmental origins of anxiety. Nat. Rev. Neurosci..

[4] Babaev O, Piletti Chatain C, Krueger-Burg D (2018). Inhibition in the amygdala anxiety circuitry. Exp. Mol. Med..

[5] Steimer T (2011). Animal models of anxiety disorders in rats and mice: some conceptual issues. Dialogues Clin. Neurosci..

[6] Fernandez SP, Gaspar P (2012). Investigating anxiety and depressive-like phenotypes in genetic mouse models of serotonin depletion. Neuropharmacology.

[7] Peca J (2011). Shank3 mutant mice display autistic-like behaviours and striatal dysfunction. Nature.

[8] Lezak KR, Missig G, Carlezon WA (2017). Behavioral methods to study anxiety in rodents. Dialogues Clin. Neurosci..

[9] Gafford GM, Ressler KJ (2016). Mouse models of fear-related disorders: cell-type-specific manipulations in amygdala. Neuroscience.

[10] Leal PC, Goes TC, da Silva LCF, Teixeira-Silva F (2017). Trait vs. state anxiety in different threatening situations. Trends Psychiatry Psychother..

[11] Zhang Y (2011). Proteomic and metabolomic profiling of a trait anxiety mouse model implicate affected pathways. Mol. Cell. Proteom..

[12] Bourin M (2015). Animal models for screening anxiolytic-like drugs: a perspective. Dialogues Clin. Neurosci..

[13] Crawley JN (1997). Behavioral phenotypes of inbred mouse strains: implications and recommendations for molecular studies. Psychopharmacology.

[14] Crawley JN, Davis LG (1982). Baseline exploratory activity predicts anxiolytic responsiveness to diazepam in five mouse strains. Brain Res. Bull..

[15] Kaidanovich-Beilin, O., Lipina, T., Vukobradovic, I., Roder, J. & Woodgett, J. R. Assessment of social interaction behaviors. *J. Vis. Exp.*10.3791/2473 (2011). 10.3791/2473 PMC3197404 21403628

[16] File SE (1980). The use of social interaction as a method for detecting anxiolytic activity of chlordiazepoxide-like drugs. J. Neurosci. Methods.

[17] Amorapanth P, LeDoux JE, Nader K (2000). Different lateral amygdala outputs mediate reactions and actions elicited by a fear-arousing stimulus. Nat. Neurosci..

[18] File SE, Seth P (2003). A review of 25 years of the social interaction test. Eur. J. Pharm..

[19] Knoll AT, Meloni EG, Thomas JB, Carroll FI, Carlezon WA (2007). Anxiolytic-like effects of kappa-opioid receptor antagonists in models of unlearned and learned fear in rats. J. Pharmacol. Exp. Ther..

[20] Knoll AT (2011). Kappa opioid receptor signaling in the basolateral amygdala regulates conditioned fear and anxiety in rats. Biol. Psychiatry.

[21] Workman JL, Trainor BC, Finy MS, Nelson RJ (2008). Inhibition of neuronal nitric oxide reduces anxiety-like responses to pair housing. Behav. Brain Res..

[22] Sani G (2012). The wnt pathway in mood disorders. Curr. Neuropharmacol..

[23] Risbrough VB, Stein MB (2006). Role of corticotropin releasing factor in anxiety disorders: a translational research perspective. Horm. Behav..

[24] Saido TC, Sorimachi H, Suzuki K (1994). Calpain: new perspectives in molecular diversity and physiological-pathological involvement. FASEB J..

[25] Castillo G (1999). An adipogenic cofactor bound by the differentiation domain of PPARgamma. EMBO J..

[26] Edelstein LC, Collins T (2005). The SCAN domain family of zinc finger transcription factors. Gene.

[27] York N (2017). Oxytocin (OXT)-stimulated inhibition of Kir7.1 activity is through PIP2-dependent Ca(2+) response of the oxytocin receptor in the retinal pigment epithelium in vitro. Cell. Signal..

[28] Smalley JL (2016). Connectivity mapping uncovers small molecules that modulate neurodegeneration in Huntington's disease models. J. Mol. Med (Berl.).

[29] Avgustinovich DF, Lipina TV, Bondar NP, Alekseyenko OV, Kudryavtseva NN (2000). Features of the genetically defined anxiety in mice. Behav. Genet..

[30] Bryant CD (2008). Behavioral differences among C57BL/6 substrains: implications for transgenic and knockout studies. J. Neurogenet..

[31] Olivier B (2002). GABAA-benzodiazepine receptor complex ligands and stress-induced hyperthermia in singly housed mice. Pharm. Biochem. Behav..

[32] Van der Heyden JA, Zethof TJ, Olivier B (1997). Stress-induced hyperthermia in singly housed mice. Physiol. Behav..

[33] Twardowschy A (2013). The role of 5-HT1A receptors in the anti-aversive effects of cannabidiol on panic attack-like behaviors evoked in the presence of the wild snake Epicrates cenchria crassus (Reptilia, Boidae). J. Psychopharmacol..

[34] Bailey, K. R. & Crawley, J. N. in *Methods of Behavior Analysis in Neuroscience Frontiers in Neuroscience* (ed Buccafusco, J. J.); CRC Press/Taylor & Francis (2009). 21204335

[35] Born G (2014). Modulation of synaptic function through the alpha-neurexin-specific ligand neurexophilin-1. Proc. Natl Acad. Sci. USA.

[36] Jones KJ (2018). Rapid, experience-dependent translation of neurogranin enables memory encoding. Proc. Natl Acad. Sci. USA.

[37] Sudhof TC (2017). Synaptic neurexin complexes: a molecular code for the logic of neural circuits. Cell.

[38] Mukherjee J (2017). Estradiol modulates the efficacy of synaptic inhibition by decreasing the dwell time of GABAA receptors at inhibitory synapses. Proc. Natl Acad. Sci. USA.

[39] Woolley SM, Gill PR, Theunissen FE (2006). Stimulus-dependent auditory tuning results in synchronous population coding of vocalizations in the songbird midbrain. J. Neurosci..

[40] Gudelsky GA, Nansel DD, Porter JC (1981). Role of estrogen in the dopaminergic control of prolactin secretion. Endocrinology.

[41] Gudelsky GA, Nansel DD, Porter JC (1981). Dopaminergic control of prolactin secretion in the aging male rat. Brain Res..

[42] Cabrera-Reyes EA, Limon-Morales O, Rivero-Segura NA, Camacho-Arroyo I, Cerbon M (2017). Prolactin function and putative expression in the brain. Endocrine.

[43] Schilling J (2015). Machine learning reveals sex-specific 17beta-estradiol-responsive expression patterns in white perch (Morone americana) plasma proteins. Proteomics.

[44] Masiero M (2013). A core human primary tumor angiogenesis signature identifies the endothelial orphan receptor ELTD1 as a key regulator of angiogenesis. Cancer Cell.

[45] Crawley J, Goodwin FK (1980). Preliminary report of a simple animal behavior model for the anxiolytic effects of benzodiazepines. Pharm. Biochem. Behav..

[46] Kechin A, Boyarskikh U, Kel A, Filipenko M (2017). cutPrimers: a new tool for accurate cutting of primers from reads of targeted next generation sequencing. J. Comput. Biol..

[47] Dobin A (2013). STAR: ultrafast universal RNA-seq aligner. Bioinformatics.

[48] Patro R, Duggal G, Love MI, Irizarry RA, Kingsford C (2017). Salmon provides fast and bias-aware quantification of transcript expression. Nat. Methods.

[49] Soneson C, Love MI, Robinson MD (2015). Differential analyses for RNA-seq: transcript-level estimates improve gene-level inferences. F1000Res.

[50] Love MI, Huber W, Anders S (2014). Moderated estimation of fold change and dispersion for RNA-seq data with DESeq2. Genome Biol..

[51] Lamb J (2007). The Connectivity Map: a new tool for biomedical research. Nat. Rev. Cancer.

[52] Lamb J (2006). The Connectivity Map: using gene-expression signatures to connect small molecules, genes, and disease. Science.

