# Supplementary material for: A computationally inspired in-vivo approach identifies a link between amygdalar transcriptional heterogeneity, socialization and anxiety
Source: Transl Psychiatry. 2019 Dec 9;9:336. doi: 10.1038/s41398-019-0677-1 (PMC6901550; doi:10.1038/s41398-019-0677-1)
Supplement: Supplementary file 1 — Supplemental Figure Legends [file 41398_2019_677_MOESM1_ESM.docx]

**Supplemental information**

Suppl. Fig. 1 Bilateral amygdalar resections

Suppl. Fig. 2 The PCA which indicates sample 5 is a clear outlier

Suppl. Fig. 3: Principal component analysis of behavior metrics.

Suppl. Fig. 4: The negative relationship between PC1 and box:zone ratios from the SIT test

Suppl. Fig. 5: Multi-region brain expression profiles of ‘top hits’ from the IPA canonical pathway (Table S2). A) Normalized expression (NX) from RNA seq obtained from the Human Protein Atlas. “Top hits” from gene expression (GNAI2, EGR4, CLTB, RPS6KB2, POMC and CACNA1A) were input into the Human Protein Atlas to analyze expression in multiple regions of human and mouse brain. Human gene expression represents the consensus normalized expression (NX) by combining the data from two transcriptomics datasets (GTEx and FANTOM5). Mouse RNA seq. based on the HPA RNA seq. tissue data (Human Protein Atlas). B) The expression values of POMC based on in situ hybridization (ISH) available through the 2004 Allen Institute for Brain Science, Allen Mouse Brain Atlas).

Table S1. Gene expression profiles and associated nomenclature for the top and bottom de-regulated genes in ‘anxious’ vs. ‘non-anxious’ C57/BL6.

Table S2. The 202 ‘anxiety’ gene signature was analyzed using IPA core analysis. The genes were matched to proteins in the IPA canonical pathway database and p-values generated based on the degree of representation relative to the number of proteins in the canonical pathway. Only pathways with p<0.05 are displayed with 4 or more genes identified in the pathway.

Table S3. The 202 ‘anxiety’ gene signature was compared to 1.3 million gene expression profiles in the cMap database. The top 15 most similar perturbagens were identified based on the number of matching signatures.
