# Supplementary figures and images for: A computationally inspired in-vivo approach identifies a link between amygdalar transcriptional heterogeneity, socialization and anxiety
Source: Transl Psychiatry. 2019 Dec 9;9:336. doi: 10.1038/s41398-019-0677-1 (PMC6901550; doi:10.1038/s41398-019-0677-1)

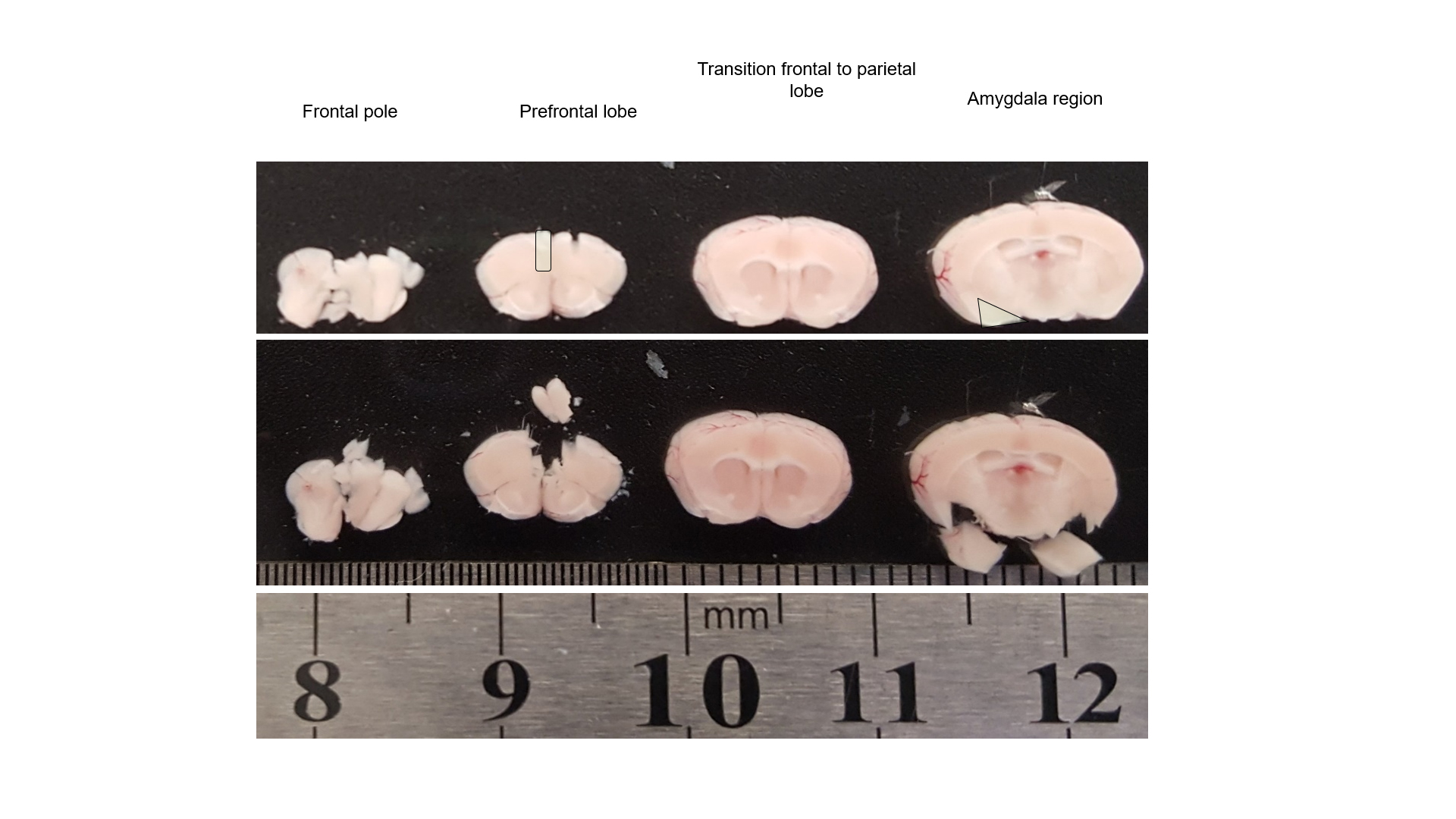

Supplement: Supplementary file 2 — Supplemental Figure 1 [file 41398_2019_677_MOESM2_ESM.jpg]

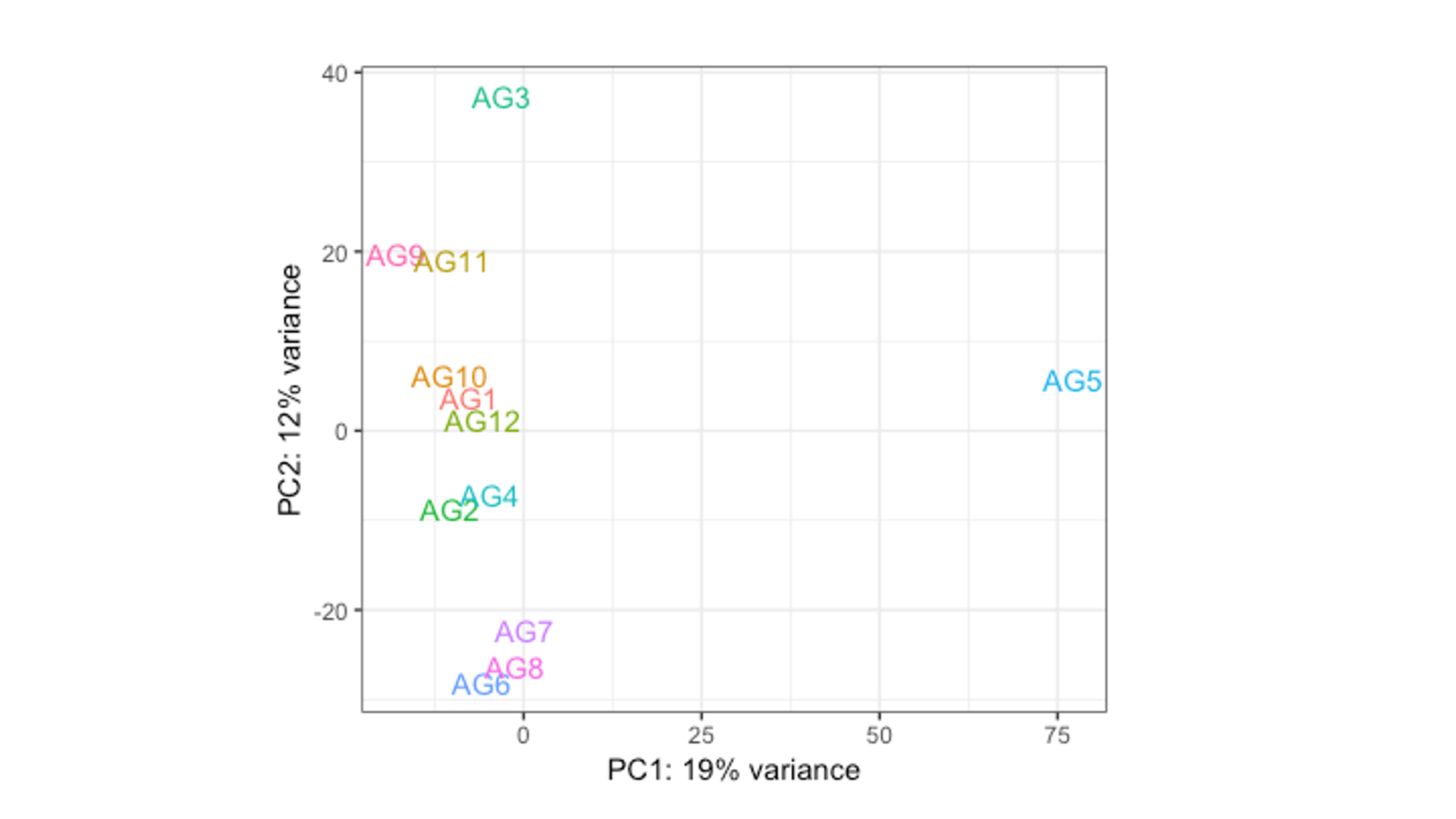

Supplement: Supplementary file 3 — Supplemental Figure 2 [file 41398_2019_677_MOESM3_ESM.jpg]

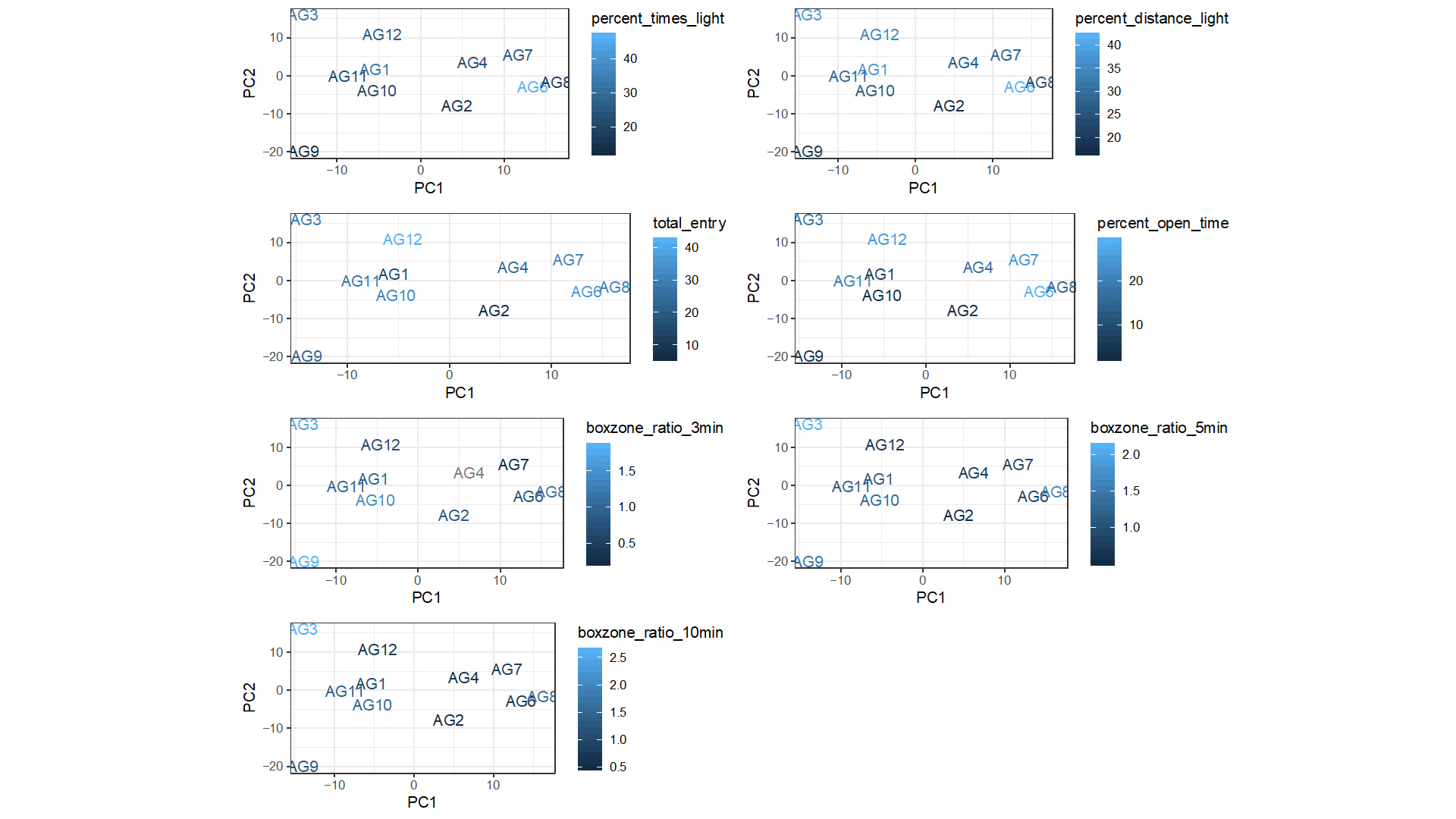

Supplement: Supplementary file 4 — Supplemental Figure 3 [file 41398_2019_677_MOESM4_ESM.jpg]

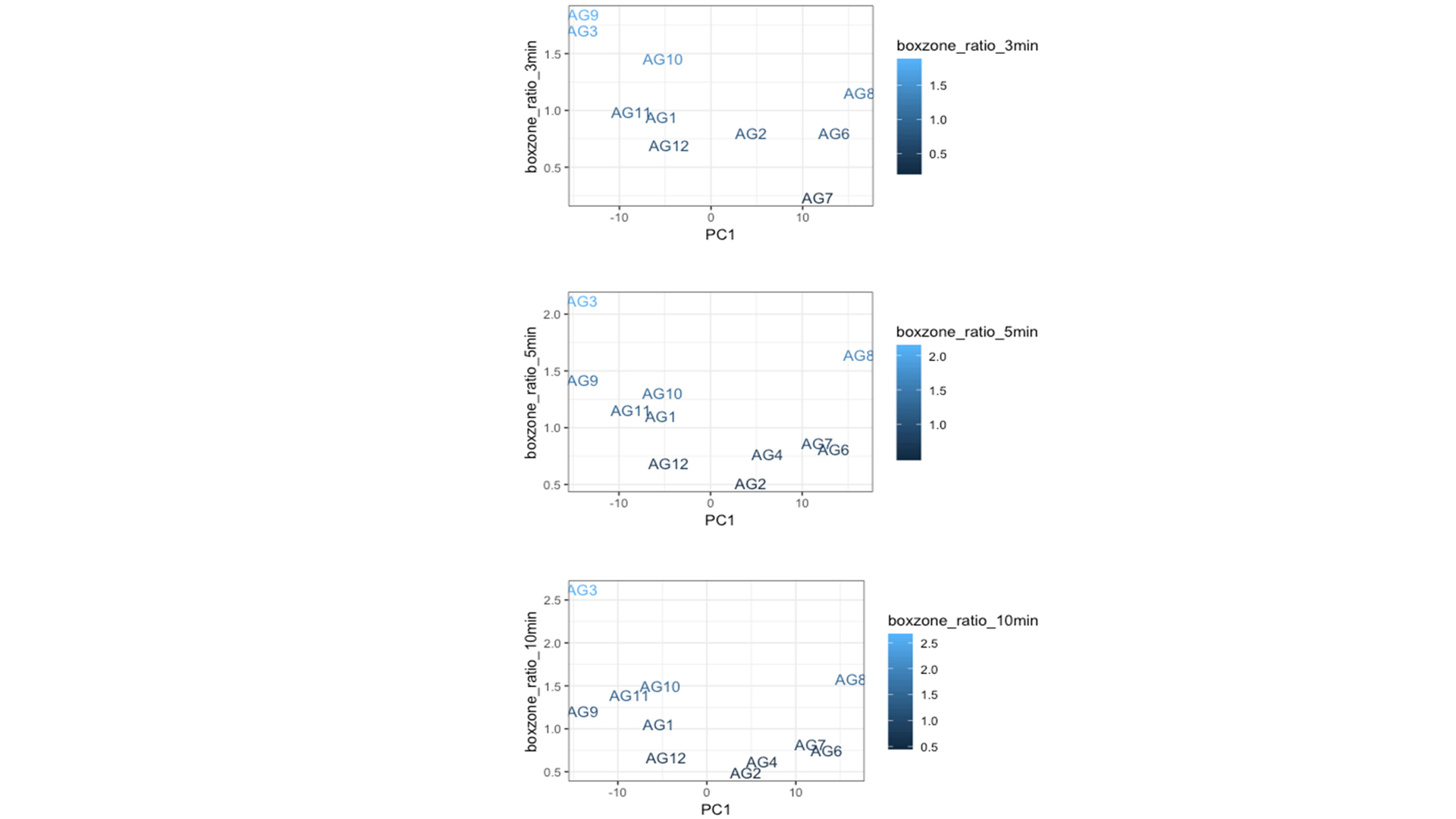

Supplement: Supplementary file 5 — Supplemental Figure 4 [file 41398_2019_677_MOESM5_ESM.jpg]

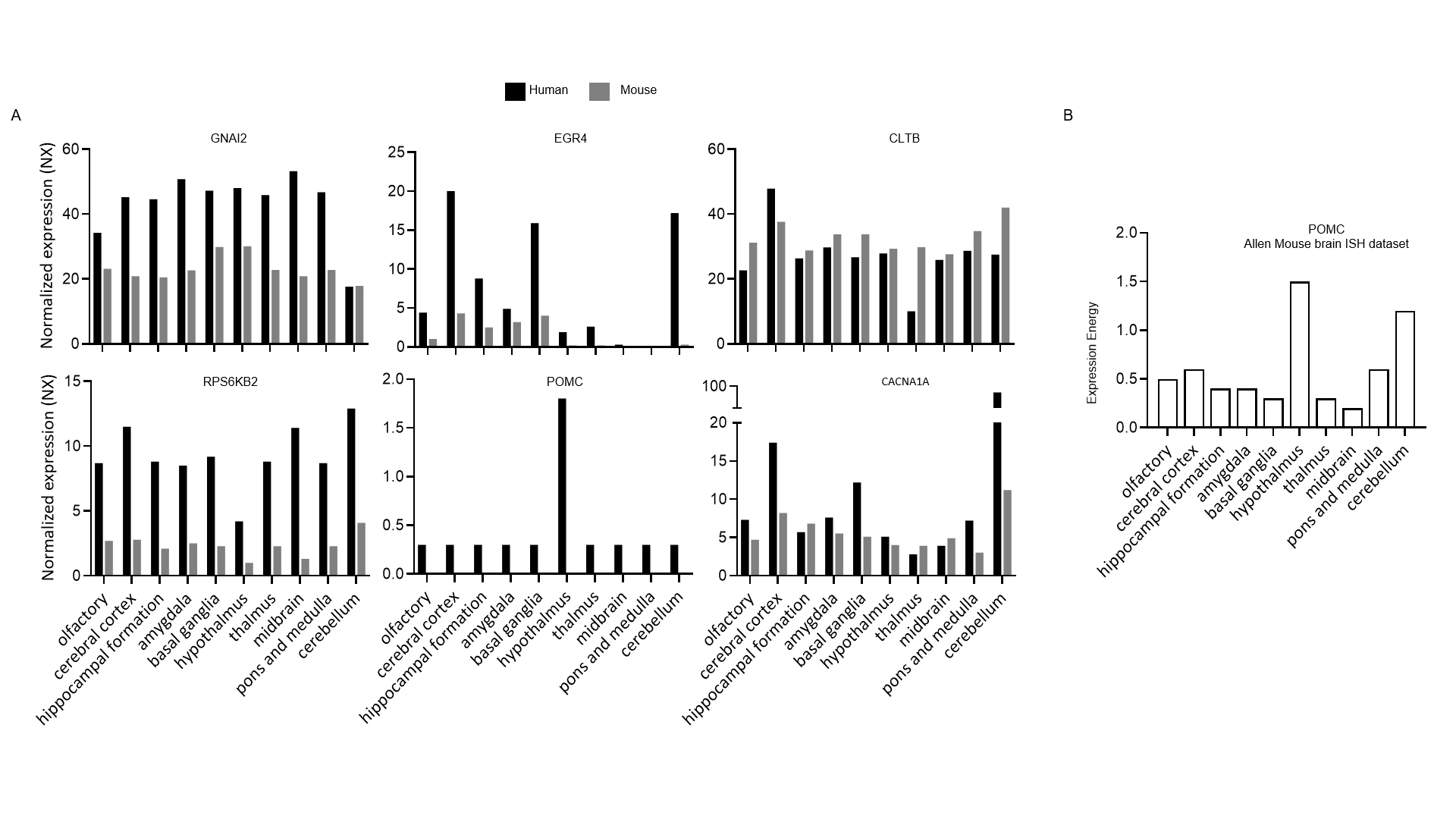

Supplement: Supplementary file 6 — Supplemental Figure 5 [file 41398_2019_677_MOESM6_ESM.jpg]
